# Host plant density and patch isolation drive occupancy and abundance at a butterfly's northern range margin

**DOI:** 10.1002/ece3.2597

**Published:** 2016-12-20

**Authors:** Yoan Fourcade, Erik Öckinger

**Affiliations:** ^1^ Department of EcologySwedish University of Agricultural SciencesUppsalaSweden

**Keywords:** climate change, habitat quality, land‐use, metapopulation, microclimate, peripheral populations

## Abstract

Marginal populations are usually small, fragmented, and vulnerable to extinction, which makes them particularly interesting from a conservation point of view. They are also the starting point of range shifts that result from climate change, through a process involving colonization of newly suitable sites at the cool margin of species distributions. Hence, understanding the processes that drive demography and distribution at high‐latitude populations is essential to forecast the response of species to global changes. We investigated the relative importance of solar irradiance (as a proxy for microclimate), habitat quality, and connectivity on occupancy, abundance, and population stability at the northern range margin of the Oberthür's grizzled skipper butterfly *Pyrgus armoricanus*. For this purpose, butterfly abundance was surveyed in a habitat network consisting of 50 habitat patches over 12 years. We found that occupancy and abundance (average and variability) were mostly influenced by the density of host plants and the spatial isolation of patches, while solar irradiance and grazing frequency had only an effect on patch occupancy. Knowing that the distribution of host plants extends further north, we hypothesize that the actual variable limiting the northern distribution of *P. armoricanus* might be its dispersal capacity that prevents it from reaching more northern habitat patches. The persistence of this metapopulation in the face of global changes will thus be fundamentally linked to the maintenance of an efficient network of habitats.

## 1 Introduction

Populations located at the periphery of species’ ranges have a particular value for conservation (Lesica & Allendorf, 1995). The abundance of a species typically decreases toward the edge of its range (Brown, 1984; Brussard, 1984; but see Sagarin & Gaines, 2002). Also, peripheral populations can typically only occupy a fraction of the potential habitat due to climatic constraints, resulting in more fragmented populations at the periphery than at the core of the range (Thomas, 1993). In combination, this makes marginal populations more prone to extinction (Hardie & Hutchings, 2010). In addition, populations living at the latitudinal margins of a species’ range are critical in the process of species response to climate change. Leading‐edge populations, being located at the potential colonization front, are those on which the capacity for a species to shift or expand its distribution relies (Hampe & Petit, 2005; Thuiller et al., 2008). Providing that all other requirements are met, range expansion can occur by recurrent poleward dispersal events from these populations followed by population growth in newly colonized sites (Hampe & Petit, 2005; Smale & Wernberg, 2013). Therefore, identifying the drivers of dynamics of high‐latitude populations is essential to understand the factors that shape range limits and to forecast the response of species to climate change. Ultimately, this knowledge is crucial to inform the conservation of these vulnerable populations.

Beyond climate, several biotic and abiotic factors have the potential to drive population processes at latitudinal range margins and to determine the limits of species’ ranges (Sexton, McIntyre, Angert, & Rice, 2009). For example, although there is ample evidence that many species are currently responding to climate change by shifting their distribution poleward (Chen, Hill, Ohlemuller, Roy, & Thomas, 2011; Gillings, Balmer, & Fuller, 2015; Hickling, Roy, Hill, Fox, & Thomas, 2006; Parmesan et al., 1999), the actual patterns of range shifts have been shown to result from a complex interaction between climate, biotic interactions (Van der Putten, Macel, & Visser, 2010), intrinsic species traits, and anthropogenic pressures (Jetz, Wilcove, & Dobson, 2007). In this regard, habitat fragmentation caused by human land use can be a key limiting factor. It may significantly impact range shift opportunities (Burrows et al., 2014) and can accelerate the extinction of isolated populations by disconnecting them from other suitable areas (Opdam & Wascher, 2004). Local habitat quality can also interact with climate variables in determining range boundaries and the response of populations to climate change (Kleijn et al., 2010; Nicolè, Dahlgren, Vivat, Till‐Bottraud, & Ehrlén, 2011; Seabrook et al., 2014). Biotic interactors (prey, predators, or hosts) are important determinants of habitat quality and can strongly influence population performance and species distribution (Louthan, Doak, & Angert, 2015). They can be so important that a mismatched response to climate change can limit range shifts that would have otherwise occurred if species tracked solely their climate niche (Pelini et al., 2009; Schweiger, Settele, Kudrna, Klotz, & Kuhn, 2008).

One approach to determine the relative importance of these factors is to study their impact on the dynamics of high‐latitude populations. In this regard, population responses to different microclimates offer an indirect but useful assessment of the climate‐related reaction norm of the species (Lawson, Bennie, Hodgson, Thomas, & Wilson, 2014; Thomas, 1993). If microclimate is the main driver of the abundance or distribution of these marginal populations, it would suggest that the species’ range is likely limited by climatic factors. We can thus expect the species to react to climate change by shifting its range poleward (Bennie et al., 2013). If, instead, habitat fragmentation is already limiting current patterns, restoring landscape connectivity may be pivotal to the conservation of these populations. Otherwise, it is unlikely that the species will be able to cope with climate change and expand its range unless new habitats are created. Similarly, if population demography and distribution at leading‐edge margins closely depend on such biotic factors, the response of the focal species to future changes will largely be driven by the response of its co‐occurring species (Gilman, Urban, Tewksbury, Gilchrist, & Holt, 2010; Van der Putten et al., 2010). Whether these populations can be the starting point of climate change tracking thus depends on their fine‐scale drivers of distribution and dynamics and on the species intrinsic habitat requirements.

**Figure 1:**
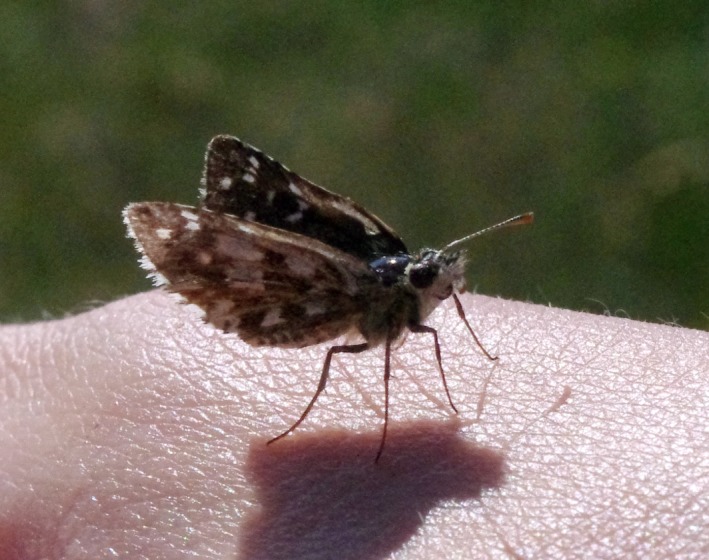
Adult Oberthür's grizzled skipper (*Pyrgus armoricanus*). Photograph by Theresia Widhalm and Alexander Neubauer

Here, we examined the effect of variation in solar irradiance (used as a proxy for potential microclimate), patch quality, or connectivity across a network of patches at a butterfly's northern range margin. We used a habitat network including a large part of the total population of Oberthür's grizzled skipper (*Pyrgus armoricanus*) (Figure 1) in Sweden as a model to infer the importance of microclimate, patch quality, and connectivity on the regional distribution and local abundance at a species’ northern range margin. The dynamics of *P. armoricanus* in southern Sweden has been proposed to be driven by metapopulation processes (Öckinger, 2006). Following metapopulation theory (Hanski, 1998), we expect the spatial configuration of patches—their area and their degree of isolation from the surrounding patches—to be an important driver of *P. armoricanus* abundance and habitat occupancy. This would highlight the importance of dispersal opportunities and thus the decisive impact of human land use on the current conservation status of the species and on its future response to climate change. Similarly, as vegetation structure is known to affect butterflies in general (Kruess & Tscharntke, 2002), and this species in particular (Eilers, Pettersson, & Öckinger, 2013), we also investigated the effect of variable grazing intensities on interpatches variation in occupancy and abundance. Moreover, we know from a previous study that microclimate influences the choice of oviposition sites in this species (Eilers et al., 2013). This factor is thus likely to affect the observed probability of habitat patches to be occupied and the population size they can sustain. Finally, owing to the fact that the presence of *P. armoricanus* in a grassland patch is generally closely linked to the availability of its host plant species (Öckinger, 2006), we also assessed the effect of the density of hosts. By ranking the importance of each of these factors in explaining variation in occupancy and abundance among habitat patches, we can gain insights into the population processes acting at high‐latitude range margins and more specifically the potential response of this species to climate change.

## 2 Methods

### 2.1 Study area and data collection

**Figure 2:**
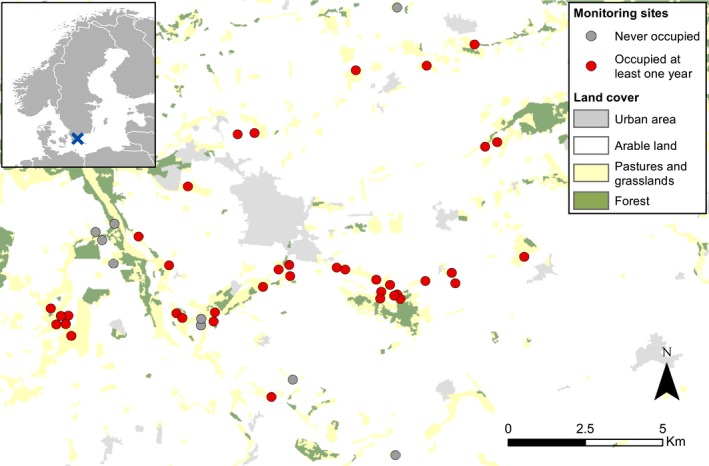
Location of the study area (shown as a cross in upper left inset) and spatial configuration of the 50 sites surveyed in this study

Our study species, Oberthür's grizzled skipper (*P. armoricanus*) (Figure 1), has a wide but fragmented distribution throughout North Africa and Europe. Its northernmost populations are located in southern Scandinavia (Sweden and Denmark), in a relative isolation from other populations in western and central Europe (Kudrna et al., 2011). Its habitat consists of seminatural grasslands which host the specific plant species where it lays its eggs and on which its larvae feed. Scandinavian populations are known to select primarily *Filipendula vulgaris* and *Helianthemum nummularium* (Christensen, 2000; Eilers et al., 2013), two species that are known to occur in a fragmented distribution up to 600 km further north (Hultén, 1971). The species has two generations per year: Spring generation adults fly from mid‐ or late May to mid‐June, and the summer generation flies in August. In addition to Denmark, its Scandinavian distribution is restricted to a small area of ca. 30 × 20 km in southern Sweden where it occurs in a network of small and fragmented patches (Öckinger, 2007). There exist no records of historical occurrences further north (Eliasson, Ryrholm, Gärdenfors, Holmer, & Jilg, 2005; Nordström, Opheim, & Valle, 1955). The habitat patches analyzed in this study (min area: 0.028 ha, max area: 14.71 ha), defined as patches of dry unfertilized grasslands with the presence of at least one of the host plants *F. vulgaris* and *H. nummularium* (Öckinger, 2006), were located in the core of this system, mainly around the town of Tomelilla (Figure 2). Adjacent habitat patches were defined as discrete if separated by at least 50 m of divergent vegetation (often arable fields or agriculturally improved grassland) as recommended by Ojanen, Nieminen, Meyke, Poyry, and Hanski (2013).

The occurrence and abundance of *P. armoricanus* were monitored in 50 habitat patches from 2004 to 2015. Between 2004 and 2011, sites were surveyed twice a year to record the abundance of both generations. From 2012, only the summer generation was surveyed. Of the 50 patches, 34 were surveyed all 12 years, eight sites 11 years, six during 9 years, one during 8 years, and one site during 7 years only. Sites were monitored by slowly walking a transect (10 m width) covering the entire area of the patch. As transects were designed to allow the entire patch area to be surveyed, the transect length was proportional to patch area, but, the sampling effort per unit area was constant. Counts should thus reflect butterfly abundance within patches and not only transect length. All observed adult *P. armoricanus* individuals were recorded. If necessary, species identity was confirmed by capturing individuals with a handheld net. As we observed that the abundance of the summer generation is on average almost three times higher than the spring generation (Table 1), we considered that processes acting in each of them might be different (see, for example, Roy & Thomas, 2003) and thus analyzed each generation separately. Therefore, we derived for each habitat patch and each generation: occupancy and the average and variability of abundance. Occupancy was expressed, separately for spring and summer generations, as the number of years a patch was occupied divided by the number of years it was surveyed in this generation. As imperfect detection can bias estimates of butterfly abundance and occupancy, it is sometimes advised to use multiple surveys per season to accurately estimate occupancy rates (MacKenzie et al., 2002). Instead, we chose here, due to time limitations, to maximize the number of patches visited each year rather than visiting each patch multiple times. However, we accounted for possible incomplete detection by considering a patch unoccupied only when no individuals were recorded during two consecutive surveys, whether these surveys occurred in the same year (spring and summer generations) or in different years (summer generation at year t and spring or summer generation—when only summer generations were surveyed—at year *t* + 1). This conservative estimate should ensure the robustness of our results even if some sites were mistakenly assumed to be unoccupied. Abundance was measured only for patches that have been recorded as occupied in at least one survey, regardless of the generation. This means that, for example, if a patch was only occupied in one summer generation survey, it was still retained for the abundance analyses of the spring generations, where it thus had a mean abundance of zero. Abundance variability was defined as the coefficient of variation of abundances across years.

**Table 1:** Average characteristics of the 50 sites surveyed. Response variables: mean (±*SD*) occupancy, average, and coefficient of variation (CV) of abundance per patch for each generation. Explanatory variables: mean (±*SD*) connectivity (depends on abundance of other patches, hence one value for each generation), area, host plant density, mean and standard deviation of solar irradiance per site, and number of sites in each category of grazing

	Spring generation	Summer generation
Dependent variables		
Occupancy	0.47 (±0.39)	0.52 (±0.40)
Average abundance^a^	6.13 (±10.39)	16.16 (±27.83)
CV abundance^a^	1.53 (±0.59)	1.62 (±0.73)
Explanatory variables		
Connectivity	4.46 (±7.87)	11.80 (±19.41)
Area (m^2^)	15,756 (±24,882)	
Host plant density (%)	3.61 (±5.63)	
Grazing		
Never	7	
Sometimes	19	
Always	24	
Mean solar irradiance (Wh/m^2^)	807,542 (±48,325)	
*SD* solar irradiance (Wh/m^2^)	47,678 (±31,754)	

a Excluding sites that were never occupied during the 12 years of survey.

We characterized each habitat patch by six variables that described habitat quality, solar irradiance, or spatial configuration of patches (Table 1). In each habitat patch, we estimated the density of *F. vulgaris* and *H. nummularium*. This was done by randomly placing 10 quadratic 1 m^2^ plots along the butterfly monitoring transect, and estimating the percentage cover of each host plant separately within each plot. Densities of host plants and flowers were recorded in May–June 2010. As both host plants are perennial and their densities have remained relatively stable over time (E. Öckinger, personal observation), the records from 2010 are assumed to represent the entire study period. *P. armoricanus* females show similar preferences for *F. vulgaris* and *H. nummularium*, although the former plant species is typically much more abundant (Eilers et al., 2013). Therefore, we pooled the cover of *F. vulgaris* and *H. nummularium* and used the averaged value over the 10 plots as a measure of host plant density per patch. All patches had a density of host plants between 0% and 15%, except one habitat patch that showed an exceptionally high host plant density of ca. 34%. We also categorized each patch by its grazing frequency according to three classes: sites that were never grazed during the whole period of survey (thereafter referred to as “never”), sites that were grazed every year (“always”) and sites that were grazed or not depending on the year (“sometimes”). We used solar irradiance to characterize potential microclimate, a feature that is known to affect *P. armoricanus* oviposition site selection (Eilers et al., 2013). Solar irradiance depends on latitude, elevation, aspect, slope, and surrounding topography and reflects the level of energy that is received at a given point of Earth. We used a digital elevation model from the Swedish Lantmäteriet (2015), produced by laser scanning. The accuracy of the elevation model is 0.5 m, and the resolution of grid cells is 2 m. Insolation was estimated as the total direct solar irradiance per 2 m grid cell per year (Wh/m^2^), using the solar radiation function in the Spatial Analyst toolbox in ArcGIS 10.2 (ESRI Inc., Redlands, CA, USA), based on latitude, slope, aspect, and effects of shading from the elevations of surrounding cells. We calculated the mean and standard deviation of solar irradiance for each habitat patch to represent the average and variability in microclimate.

We also quantified for each patch the two main predictors of metapopulation dynamics: area and connectivity. The boundaries of habitat patches were defined in the field and digitized in a GIS. To account for the fact that transect lengths were dependent on patch area (see monitoring protocol above), we included area as a covariable in all analyses. Patch areas were calculated using ArcGIS 10.2. As a measure of connectivity, we used the index *S*_*i*_ developed by Hanski (1999) and calculated as $S_{i}=\sum_{j\neq i}e^{-\alpha d_{ij}}N_{j}$. *S*_*i*_ estimates the connectivity at patch *i*, where *d*_*ij*_ is the Euclidian distance between patches *i* and *j* (in meters) and *N*_*j*_ the observed abundance at patch *j* (reflecting the number of potential emigrants from patch *j*), α being a constant describing how fast immigration probability from patch *j* decreases with increasing distance. Here, α was set as 0.0034, corresponding to an average movement distance of 295 m, according to a previous analysis of mark–recapture data (E. Öckinger, unpublished data). When calculating connectivity, we took into account not only the 41 patches analyzed in this study, but all potential habitat patches within a 35 × 35 km square including the entire known distribution of *P. armoricanus* in Sweden (25 patches in addition to the 50 surveyed patches). As this index depends on abundance of all surrounding patches, we calculated *S*_*i*_ for each year and generation, and used in further analyses its average value over all years for each generation separately. When abundance data were unavailable in a given year, we used the average abundance at this site across all years of survey. Similarly, for all sites that were not part of the annual monitoring, we used a rough estimation of abundance at these sites based on a systematic mapping of the species’ entire distribution in Sweden in 2007 and 2010.

### 2.2 Statistical analyses

We analyzed the effect of habitat quality, microclimate, area, and connectivity on patch occupancy and the average and variability of butterfly abundance in both generations separately. Occupancy was analyzed by a generalized linear model with binomial error distribution and logit link. The response variable was defined, for each generation, as the number of years a patch was occupied divided by the number of years it was surveyed. To account for potentially false absences, we took a conservative approach and only considered a patch as unoccupied if no *P. armoricanus* individuals were observed there during two consecutive surveyed generations; otherwise, the patch was considered as being still occupied. We used linear regressions to model average and variability of abundance per site. The average and coefficient of variability of abundance were log‐transformed prior to analyses so that model predictions fall in the interval [0, +∞]. Moreover, a small value (0.1) was added to all abundance data so that the sites that were never occupied could be included as well (log‐transformation impossible when average abundance = 0). As explanatory predictors, we used two variables linked to the spatial configuration of sites: patch area and connectivity, two variables describing habitat quality: host plant density and grazing frequency (defined as three categories: always, never, or sometimes grazed during the survey period), and two variables describing microclimate: mean and standard deviation of solar irradiance. In addition, we aimed to test whether the proximity of neighboring patches, and thus a higher immigration probability in a metapopulation model, could balance low habitat quality or unfavorable microclimatic conditions. Therefore, we also included in all models as explanatory variables the two‐way interactions between connectivity and site quality variables (host plant density and grazing) or microclimate (mean and *SD* of solar irradiance), resulting in four additional predictors.

We adopted an information‐theoretic approach (Burnham & Anderson, 2002) by computing models with all combinations of variables, and ranked them by their second‐order Akaike information criterion (AIC_c_). A multimodel inference was then performed by averaging all models whose cumulative Akaike weight was <0.95 (Burnham & Anderson, 2002). We extracted standardized averaged parameter estimates of all variables and interactions and estimated relative variable importance based on the sum of Akaike weights of all candidate models containing the variable. Multimodel inferences were run using the “MuMIn” package (Barton, 2013) in R 3.2.2 (R Development Core Team, 2015). Partial relationships were visualized by plotting model predictions against variations in the variable of interest while holding all other variables at their median—grazing frequency being set to “sometimes.” For visualizing interactions with connectivity, this approach was performed for three levels of connectivity corresponding to its 0.25, 0.5, and 0.75 quantiles.

## 3 Results

Nine patches were occupied through all 12 years, nine patches were never occupied, and 32 patches were occupied during at least 1 year. Patch occupancy thus ranged from 0 to 1 (mean = 0.51 ± 0.39 *SD*) when both generations where considered together, with roughly similar values in the spring (from 0 to 1, mean = 0.47 ± 0.39 *SD*) and the summer generations (from 0 to 1, mean = 0.52 ± 0.40 *SD*) (Appendix 1 and Table 1). The average abundance per patch (both generations: from 0.053 to 98.05, mean = 12.50 ± 21.34 *SD*) was generally higher during the summer generation (from 0 to 128.75; mean = 16.16 ± 27.83 *SD*) than during the spring generation (from 0 to 45.43; mean = 6.13 ± 10.39 *SD*) (Appendix 1 and Table 1), which motivated the analyses of each generation separately. Despite that, the coefficient of variation of abundance (both generations: from 0.75 to 4.36, mean = 1.86 ± 0.90 *SD*) was largely similar between generations (0.52–2.65, mean = 1.53 ± 0.59 *SD* for the spring generation, and 0.61–3.46, mean = 1.62 ± 0.73 *SD* for the summer generation) (Appendix 1 and Table 1).

**Figure 3:**
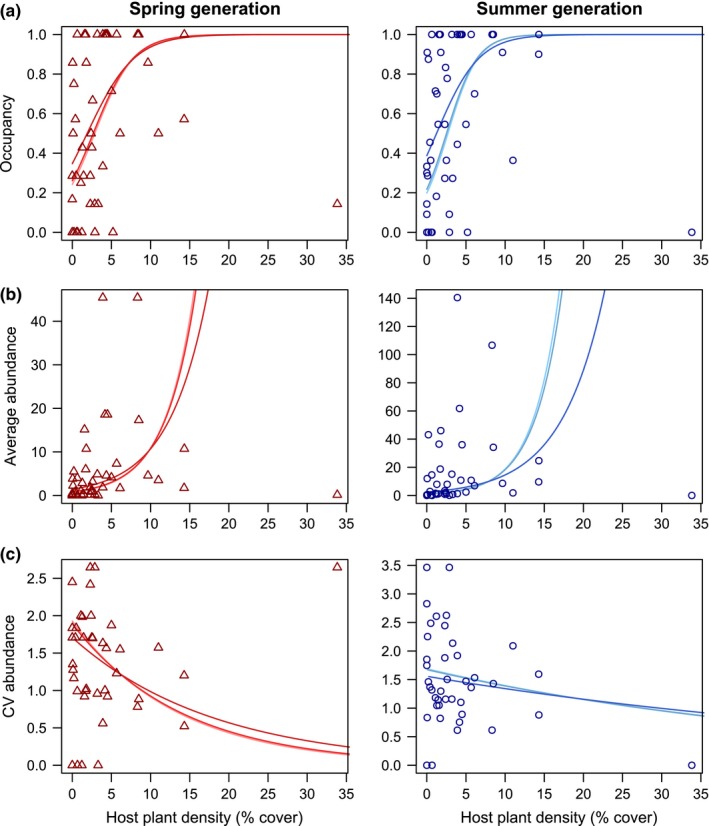
Partial relationships showing model averaging predictions for (a) occupancy, (b) average, and (c) coefficient of variation of abundance across the range of host plant density observed in the patches. As models included the interaction between host plant density and connectivity, we plotted predictions for three levels of connectivity corresponding to 0.25, 0.5, and 0.75 quantiles (connectivity increases from lighter to darker colors). Plots are shown for models built with spring (red, left) and summer generation (blue, right) data, and observed values are displayed as triangles and circles, respectively

Results for the models explaining variation in occupancy among patches were generally similar between generations (Table 2 and Figure 3a). For both generations, the most important variables were patch area, connectivity, grazing frequency, and host plant density which all were positively related with occupancy and had relative importance = 1. Solar irradiance (mean and *SD*) also had a high relative importance in the models, especially in the summer generation (importance > 0.8), and revealed that patches with a higher and more variable microclimate were occupied more frequently. The interaction between connectivity and host plant density (importance = 0. 83 and 1 for spring and summer generations, respectively) implies that, while occupancy generally increased with the density of host plants or with connectivity, host plant density had the largest effect in the most isolated patches, and conversely, connectivity had the largest effect on occupancy in patches with a low density of hosts (Figure 3a). Averaged parameter estimates showed that the effect of patch area (coefficient = 0. 61 and 0.51 for spring and summer generations), host plant density (0.85 and 0.72), and the interaction between host plant density and connectivity (−0.70 and −0.84) largely exceeded that of other variables (all other absolute coefficients < 0.5).

**Table 2:** Ninety‐five percent model‐averaged coefficients (±*SE*) and variable importance from linear models explaining for each generation (a) occupancy, (b) average, and (c) coefficient of variation of abundance per patch. For grazing regime, the “never” category is taken as reference. Variable importance > 0.5 is highlighted in bold font, and the three highest absolute coefficient value for each model is displayed in italic font

Variables	Spring generation			Summer generation		
	Estimate	*SE*	Importance	Estimate	*SE*	Importance
(a) Occupancy						
Area	*0.621*	0.181	**1.000**	*0.512*	0.113	**1.000**
Connectivity	0.255	1.170	**1.000**	0.393	0.724	**1.000**
Grazing (sometimes)	0.405	0.127	**1.000**	0.193	0.057	**1.000**
Grazing (always)	0.244	0.141		0.136	0.062	
Host plant density	*0.852*	0.146	**1.000**	*0.717*	0.103	**1.000**
Solar irradiance (mean)	0.049	0.074	0.453	0.121	0.040	**1.000**
Solar irradiance (*SD*)	0.007	0.049	0.252	0.072	0.055	**0.823**
Connectivity:Grazing (sometimes)	0.122	0.353	**0.725**	0.050	0.156	0.324
Connectivity:Grazing (always)	0.400	0.515		0.120	0.250	
Connectivity:Host plant density	−*0.697*	0.431	**0.828**	−*0.839*	0.225	**1.000**
Connectivity:Solar irradiance (mean)	−0.206	0.885	0.105	−0.239	0.626	0.266
Connectivity:Solar irradiance (*SD*)	0.020	0.084	0.075	0.032	0.067	0.293
(b) Average abundance						
Area	*0.314*	0.168	**0.905**	*0.441*	0.127	**1.000**
Connectivity	0.121	1.140	**1.000**	0.200	0.936	**1.000**
Grazing (sometimes)	0.252	0.290	**0.578**	0.105	0.202	0.315
Grazing (always)	0.102	0.223		0.063	0.170	
Host plant density	*0.901*	0.264	**1.000**	*0.871*	0.242	**1.000**
Solar irradiance (mean)	0.001	0.065	0.179	−0.007	0.063	0.184
Solar irradiance (*SD*)	−0.017	0.086	0.206	0.012	0.076	0.211
Connectivity:Grazing (sometimes)	0.208	0.433	0.227	0.125	0.322	0.159
Connectivity:Grazing (always)	0.300	0.602		0.235	0.578	
Connectivity:Host plant density	−*0.939*	0.570	**0.809**	−*1.052*	0.512	**0.866**
Connectivity:Solar irradiance (mean)	0.036	0.613	0.025	0.015	0.484	0.021
Connectivity:Solar irradiance (*SD*)	0.006	0.064	0.033	0.005	0.056	0.033
(c) CV abundance						
Area	−*0.425*	0.131	**1.000**	−*0.268*	0.202	**0.779**
Connectivity	−0.036	1.458	**1.000**	−0.112	0.535	**0.522**
Grazing (sometimes)	−0.335	0.278	**0.724**	−*0.243*	0.333	0.441
Grazing (always)	−0.407	0.312		−*0.240*	0.331	
Host plant density	−*1.126*	0.263	**1.000**	−0.174	0.211	**0.569**
Solar irradiance (mean)	−0.013	0.082	0.243	−0.004	0.081	0.188
Solar irradiance (*SD*)	−0.011	0.067	0.168	−0.162	0.204	**0.600**
Connectivity:Grazing (sometimes)	−0.014	0.142	0.024	−0.013	0.238	0.026
Connectivity:Grazing (always)	−0.016	0.166		−0.031	0.330	
Connectivity:Host plant density	*1.272*	0.357	**0.987**	0.061	0.195	0.131
Connectivity:Solar irradiance (mean)	−0.321	1.405	0.079	0.005	0.313	0.006
Connectivity:Solar irradiance (*SD*)	0.001	0.032	0.012	−0.026	0.130	0.077

For average abundance, results from model averaging showed highly similar responses for both generations (Table 2 and Figure 3b). The most important variables explaining average abundance were patch area, connectivity, and host plant density (relative importance in all cases > 0.9). The interaction between connectivity and host plant density had also a high importance in the models (>0.8). As for occupancy, average abundance increased with patch area and host plant density or connectivity. Again, the interaction between the latter two revealed that highly connected patches were less sensitive to host density (Figure 3b). Grazing intensity had an intermediately high importance (variable importance = 0.58 and 0.32 for spring and summer generations, respectively), indicating that grazing allowed patches to sustain a higher population size on average compared to nongrazed sites. All other variables or interactions had a relative importance between 0.03 and 0.23. Parameter estimates pointed to a greater effect of host plant density (coefficient = 0.90 and 0.87 for spring and summer generations), especially in interaction with connectivity (−0.94 and −1.05), compared to other variables (all other absolute coefficients < 0.5).

For the variability in abundance among years, models led to some notably different responses between generations (Table 2). In the spring generation, the variability in abundance tended to be reduced in large well‐connected patches with a high host plant density (relative importance always = 1) and regularly grazed (0.72), although again the effect of plant density was reduced in highly connected patches (relative importance for interaction term = 0.99, Figure 3c). Model‐averaged coefficients revealed that host plant density, alone (coefficient = −1.13) and in interaction with connectivity (coefficient = 1.27), had the highest effects on site variability during first generation. In contrast, the relative importance was more evenly distributed among variables in the second‐generation model. Only patch area had a strong negative effect on abundance variability in the averaged model (importance = 0.78), followed by the standard deviation of solar irradiance (0.60) and host plant density (0.57) which both decreased the variability of abundance as they increased. Similarly, averaged estimates remained limited, with only area and grazing having a negative effect on site variability with a coefficient > 0.2. The full set of models used for model averaging is given in Appendix 2.

## 4 Discussion

Although limiting factors may change in space and time (Lawson, Bennie, Thomas, Hodgson, & Wilson, 2012), understanding the determinants of distribution and population dynamics at species range margins is fundamental for our ability to predict biodiversity responses to climate change. We demonstrated that the occupancy, abundance, and population variability at the northern range margin of the butterfly *P. armoricanus* are mainly driven by patch area, connectivity, and host plant density, while solar irradiance only had an impact on patch occupancy. Large and well‐connected habitat patches tended to be more often occupied, to have on average larger and more stable populations, and to display a higher abundance. The strong and consistent effects of patch area and connectivity show that the spatial configuration of habitat is the most important factor for population persistence at the climatic range margin of this butterfly. This pattern is congruent with metapopulation theory which predicts that occupancy and turnover rate are driven by patch area and site isolation (Hanski, 1994, 1998).

Several studies have highlighted the importance of microclimatic conditions for invertebrate populations, especially for populations near the climatic limits of the species (e.g., Bennie et al., 2013; Turlure, Choutt, Baguette, & Van Dyck, 2009; Wilson, Davies, & Thomas, 2010). We found that patches that received a high solar irradiance—presumably reflecting a warmer microclimate—were more frequently occupied than less sunny habitat patches, confirming previous findings that microclimate is an important aspect of habitat quality for this species (Eilers et al., 2013). In contrast, the effect of solar irradiance on abundance and population variability was negligible compared to other factors. Hence, our results suggest that some otherwise suitable habitat patches have a too cold microclimate to allow for persistence of *P. armoricanus* populations. On the other hand, where *P. armoricanus* is present, a higher solar irradiance does not result in larger populations. This could partly be because our surrogate for microclimate, that is, solar irradiance, only reflects the potential microclimate resulting from topography. The realized local temperature conditions can also be influenced by various factors such as vegetation cover or the surrounding landscape (Suggitt et al., 2011). A previous study found that the availability of host plants situated in a warm microclimate could predict local population sizes (Eilers et al., 2013). With warmer global temperatures, it is possible that this restriction to sites with a warm microclimate can be relaxed, and allow for colonization of a wider range of habitats at a regional scale, as has been observed for other butterflies at the climatic margins of their distributions (Pateman, Hill, Roy, Fox, & Thomas, 2012; Pateman, Thomas, Hayward, & Hill, 2016; Wilson et al., 2010). So far, however, this has not been observed for *P. armoricanus* in our study region.

The density of larval host plants, *F. vulgaris* and *H. nummularium,* appeared as a major driver of occupancy, abundance, and population variability. Larval host plants have long been known to play a vital role in the dynamics of butterfly populations (Hanski & Singer, 2001; Koh et al., 2004). As such, host plants’ dynamics and distribution can strongly affect butterflies’ response to climate change (Araújo & Luoto, 2007) and predicted range shifts by host plants could directly lead to changes or reductions in the distribution of herbivorous insects (Romo, Garcia‐Barros, Marquez, Moreno, & Real, 2014; Romo, Silvestre, & Munguira, 2015). On the contrary, a mismatched response between a butterfly and its host may make it unable to track climate change (Pelini et al., 2009; Schweiger et al., 2008), even if host switching has also been documented (Pateman et al., 2012; Thomas et al., 2001). Our results suggest that host plant availability is one of the most important factors limiting the local abundance and landscape‐scale distribution of *P. armoricanus*. However, at a larger spatial scale, the current distribution of *P. armoricanus* in Sweden is not limited by the distribution of its host plants, because both *F. vulgaris* and *H. nummularium* are known to occur much further north (Hultén, 1971). This leads us to believe that a potential expansion by the species to habitat further north is restricted by either microclimatic favorability or connectivity.

Connectivity had a particularly high importance in almost all models, especially in interaction with host plant density. Considering the effect of connectivity alone, our results reflect the fact that sites that are spatially isolated and surrounded by low‐abundance sites have generally a lower and more variable abundance and are less often occupied. This suggests that the current distribution of *P. armoricanus* could be constrained by a too high isolation of habitat patches further north, hence preventing their colonization due to limited dispersal. The effect of the interaction between host plant density and connectivity is also interesting; it reveals that the positive effect of host plants density on abundance or occupancy—and to a lesser extent site stability—tends to decrease as connectivity increases. It can likely be interpreted as a rescue effect (Brown & Kodric‐Brown, 1977) which allows low‐quality sites to be sustained by migration from surrounding patches and reduced population fluctuations in well‐connected patches. This property of metapopulation dynamics favors the persistence of a local population by decreasing extinction probability or by supporting population size through regular immigration events, even if the local conditions are suboptimal (Gonzalez, Lawton, Gilbert, Blackburn, & Evans‐Freke, 1998; Gotelli, 1991). Because patches are separated by unsuitable intensively managed fields, the isolation of habitats is in the present case strongly driven by agricultural practices which determine the connectivity of the habitat network and will in this respect be key to the long‐term persistence of this *P. armoricanus* population.

Our results challenge the still‐common view that latitudinal edges are limited purely by climatic factors (Pearson & Dawson, 2003; Woodward, 1990), but concurs with modern niche theory that assumes actual distributional limits to be formed by an interaction between abiotic factors (fundamental niche, mainly climate), biotic interactions (here larval host), and dispersal (Soberón, 2007; Soberon & Nakamura, 2009). Better understanding of the actual position of this population relative to the species niche could be gained by investigating more closely its climatic tolerance, for example experimentally, or by comparing processes acting in various parts of its range (Lawson et al., 2012). In conclusion, it appears that the regional distribution and abundance of the northernmost population of *P. armoricanus* are mostly dependent on the availability of habitat patches with a high density of its larval host plants and on its capacity to disperse between such habitat patches. An effective conservation management strategy for this species should thus act both at the patch and landscape scales. First, habitat quality of already suitable patches must be maintained by ensuring the continuation of extensive grazing practices that provide an adequate vegetation structure and density of host plants. Second, the network of habitat patches must be kept dense enough to allow the long‐term metapopulation viability. More generally, the persistence of many species in the face of climate change will be fundamentally linked to the maintenance of an efficient network of habitats (Hodgson, Thomas, Wintle, & Moilanen, 2009). It can be achieved by preserving the existent connectivity between habitats, but also by creating or restoring habitats to facilitate range shifts across a fragmented landscape (Hodgson, Wallis, Krishna, Cornell, & Isaac, 2016; Hodgson et al., 2011). In addition, enhancing local habitat quality at climatic range margins has been shown to be an effective alternative strategy to facilitate species expansion under climate change, as it secures vulnerable marginal populations and increases the pool of potential migrants (Lawson et al., 2012). Conservation planning should also take into account the current and future properties of the landscape matrix to assist species in tracking their favorable environmental conditions (Pearson & Dawson, 2005). Moreover, efficient actions should ideally consider the potential responses of all interacting species in an ecosystem (Walther, 2010), which makes management‐assisted climate change mitigation challenging. However, maintaining the potential of species to respond effectively to the ongoing human‐induced changes is essential for their persistence, especially for populations located at the margins of species distributions as, being generally located in suboptimal environmental conditions, they naturally have a higher extinction risk (Lesica & Allendorf, 1995).

## Conflict of Interest

None declared.

## Appendix 1

### 1.1

Frequency histograms of occupancy, average abundance, and coefficient of variation of abundance, for the spring (red) and summer (blue) generations. Overlapping areas appear in purple.

## Appendix 2

### 2.1

Models ranked by AIC_c_, explaining (a) occupancy, (b) average, and (c) coefficient of variation of abundance, for each generation. Only models used in model averaging are shown, that is, those whose cumulative weight was < 95%. Full models (including all variables and interactions) and null models (including only intercept) are also shown for comparison. Variables are abbreviated as follows: A: area; C: connectivity; HD: host plant density; G: grazing intensity; SRM: mean solar irradiance; SRSD: standard deviation of solar irradiance.

**Table:**

Variables	*df*	logLik	AIC_c_	ΔAIC_c_	ω
(a) occupancy					
Spring generation					
A + C + G + HD + C:G + C:HD	9	−79.714	181.929	0.000	.237
A + C + G + HD + SRM + C:G + C:HD	10	−78.523	182.687	0.758	.163
A + C + G + HD + C:G	8	−82.347	184.206	2.277	.076
A + C + G + HD + C:HD	7	−83.944	184.554	2.625	.064
A + C + G + HD + SRM + C:G	9	−81.238	184.976	3.047	.052
A + C + G + HD + SRSD + C:G + C:HD	10	−79.693	185.028	3.099	.050
A + C + G + HD + SRSD + C:HD + C:SRSD	9	−81.427	185.354	3.425	.043
A + C + G + HD + SRM + C:G + C:HD + C:SRM	11	−78.269	185.486	3.557	.040
A + C + G + HD + SRM + SRSD + C:G + C:HD	11	−78.485	185.916	3.988	.032
A + C + G + HD + SRM + C:HD	8	−83.303	186.117	4.188	.029
A + C + G + HD + SRSD + C:HD	8	−83.304	186.120	4.191	.029
A + C + G + HD + SRM + C:HD + C:SRM	9	−81.829	186.159	4.230	.029
A + C + G + HD + SRM + SRSD + C:HD + C:SRSD	10	−80.279	186.199	4.270	.028
A + C + G + HD + SRM + SRSD + C:HD	9	−81.948	186.395	4.466	.025
A + C + G + HD + SRM + C:G + C:SRM	10	−80.760	187.161	5.232	.017
A + C + G + HD + SRSD + C:G	9	−82.347	187.193	5.264	.017
Full model	13	−78.074	192.259	10.330	.001
Null model	1	−157.873	317.829	135.900	.000
Summer generation					
A + C + G + HD + SRM + SRSD + C:HD	9	−110.154	242.809	0.000	.251
A + C + G + HD + SRM + SRSD + C:HD + C:SRSD	10	−108.751	243.144	0.335	.212
A + C + G + HD + SRM + SRSD + C:HD + C:SRM	10	−109.388	244.416	1.608	.112
A + C + G + HD + SRM + SRSD + C:G + C:HD	11	−107.764	244.475	1.667	.109
A + C + G + HD + SRM + C:G + C:HD	10	−109.501	244.644	1.835	.100
A + C + G + HD + SRM + C:G + C:HD + C:SRM	11	−108.701	246.349	3.540	.043
A + C + G + HD + SRM + SRSD + C:HD + C:SRM + C:SRSD	11	−108.722	246.392	3.584	.042
A + C + G + HD + SRM + SRSD + C:G + C:HD + C:SRM	12	−107.277	246.987	4.179	.031
A + C + G + HD + SRM + C:HD + C:SRM	9	−112.458	247.415	4.606	.025
A + C + G + HD + SRM + SRSD + C:G + C:HD + C:SRSD	12	−107.519	247.471	4.662	.024
Full model	13	−107.274	250.660	7.851	.005
Null model	1	−234.571	471.225	228.416	.000
(b) Average abundance					
Spring generation					
A + C + HD + C:HD	6	−67.907	150.284	0.000	.234
A + C + G + HD + C:HD	8	−65.099	150.697	0.413	.190
A + C + G + HD + C:G	9	−64.114	152.035	1.751	.098
A + C + HD + SRSD + C:HD	7	−67.784	152.962	2.678	.061
A + C + HD + SRM + C:HD	7	−67.889	153.171	2.887	.055
A + C + G + HD + SRSD + C:HD	9	−65.085	153.976	3.692	.037
A + C + G + HD + SRM + C:HD	9	−65.088	153.983	3.699	.037
C + G + HD + C:HD	7	−68.541	154.475	4.191	.029
A + C + G + HD + SRSD + C:G	10	−63.791	154.916	4.632	.023
A + C + G + HD + C:G + C:HD	10	−63.873	155.080	4.796	.021
C + G + HD + C:G	8	−67.333	155.166	4.882	.020
A + C + G + HD + SRM + C:G	10	−64.088	155.509	5.225	.017
A + C + HD + SRSD + C:HD + C:SRSD	8	−67.638	155.775	5.491	.015
A + C + HD + SRM + SRSD + C:HD	8	−67.784	156.067	5.783	.013
A + C + HD + SRM + C:HD + C:SRM	8	−67.791	156.081	5.797	.013
A + C + G + HD + SRSD + C:HD + C:SRSD	10	−64.632	156.598	6.314	.010
C + HD + C:HD	5	−72.652	157.019	6.735	.008
C + G + HD + SRSD + C:G	9	−66.654	157.115	6.831	.008
C + G + HD + SRSD + C:HD	8	−68.458	157.417	7.133	.007
A + C + G + HD + SRM + SRSD + C:HD	10	−65.062	157.457	7.173	.006
C + G + HD + SRM + C:HD	8	−68.488	157.476	7.192	.006
A + C + G + HD + SRM + C:HD + C:SRM	10	−65.082	157.498	7.214	.006
C + G + HD + C:G + C:HD	9	−67.136	158.079	7.795	.005
A + C + G + HD + SRSD + C:G + C:HD	11	−63.494	158.092	7.808	.005
A + C + G + HD + SRM + C:G + C:SRM	11	−63.556	158.216	7.932	.004
C + G + HD + SRM + C:G	9	−67.328	158.462	8.178	.004
A + C + G + HD + SRSD + C:G + C:SRSD	11	−63.770	158.644	8.360	.004
A + C + G + HD + SRM + SRSD + C:G	11	−63.791	158.685	8.401	.004
A + C + G + HD + SRM + C:G + C:HD	11	−63.843	158.789	8.505	.003
C + G + HD + SRSD + C:HD + C:SRSD	9	−67.547	158.901	8.617	.003
C + HD + SRSD + C:HD	6	−72.284	159.038	8.754	.003
Null model	2	−79.305	162.927	12.643	.000
Full model	14	−62.529	169.211	18.927	.000
Summer generation					
A + C + HD + C:HD	6	−70.814	156.099	0.000	.380
A + C + HD + SRSD + C:HD	7	−70.633	158.660	2.561	.106
A + C + G + HD + C:HD	8	−69.166	158.831	2.732	.097
A + C + G + HD + C:G	9	−67.543	158.892	2.793	.094
A + C + HD + SRM + C:HD	7	−70.760	158.915	2.816	.093
A + C + HD + SRSD + C:HD + C:SRSD	8	−70.486	161.472	5.373	.026
A + C + G + HD + C:G + C:HD	10	−67.146	161.626	5.527	.024
A + C + G + HD + SRSD + C:HD	9	−68.920	161.645	5.546	.024
A + C + HD + SRM + SRSD + C:HD	8	−70.625	161.750	5.651	.023
A + C + G + HD + SRM + C:HD	9	−69.012	161.830	5.731	.022
A + C + HD + SRM + C:HD + C:SRM	8	−70.730	161.960	5.860	.020
A + C + G + HD + SRSD + C:G	10	−67.541	162.416	6.317	.016
A + C + G + HD + SRM + C:G	10	−67.543	162.419	6.320	.016
A + C + G + HD + SRSD + C:HD + C:SRSD	10	−68.621	164.575	8.476	.005
Null model	2	−85.588	175.492	19.393	.000
Full model	14	−66.340	176.834	20.735	.000
(c) coefficient of variation of abundance					
Spring generation					
A + C + G + HD + C:HD	8	−1.678	24.689	0.000	.421
A + C + HD + C:HD	6	−5.920	26.737	2.048	.151
A + C + G + HD + SRM + C:HD	9	−1.513	27.949	3.260	.083
A + C + G + HD + SRSD + C:HD	9	−1.603	28.130	3.441	.075
A + C + G + HD + SRM + C:HD + C:SRM	10	−0.148	29.096	4.407	.047
A + C + HD + SRM + C:HD	7	−5.548	29.097	4.407	.046
A + C + HD + SRSD + C:HD	7	−5.838	29.676	4.986	.035
A + C + HD + SRM + C:HD + C:SRM	8	−4.865	31.063	6.374	.017
A + C + G + HD + SRM + SRSD + C:HD	10	−1.317	31.433	6.744	.014
A + C + G + HD + C:G	9	−3.409	31.740	7.051	.012
A + C + G + HD + SRSD + C:HD + C:SRSD	10	−1.526	31.851	7.162	.012
A + C + HD + SRM + SRSD + C:HD	8	−5.261	31.856	7.167	.012
A + C + G + HD + SRM + SRSD + C:HD + C:SRM	11	0.467	32.066	7.377	.011
A + C + G + HD + C:G + C:HD	10	−1.678	32.155	7.466	.010
Null model	2	−19.095	42.554	17.865	.000
Full model	14	1.620	44.760	20.071	.000
Summer generation					
A + SRSD	4	−17.826	44.864	0.000	.064
A + G + HD + SRSD	7	−13.711	45.155	0.292	.055
A + C + SRSD	5	−16.669	45.213	0.349	.054
A + HD + SRSD	5	−16.922	45.719	0.856	.042
A + G + HD	6	−15.639	45.988	1.124	.036
A + G + SRSD	6	−15.702	46.113	1.249	.034
A + C	4	−18.574	46.361	1.497	.030
A + C + HD + C:HD	6	−15.853	46.415	1.551	.029
A + C + HD + SRSD	6	−16.036	46.781	1.917	.024
A + C + HD	5	−17.540	46.954	2.090	.022
A + HD	4	−18.970	47.152	2.288	.020
A + C + G + HD + C:HD	8	−13.104	47.174	2.310	.020
G + HD	5	−17.694	47.262	2.398	.019
A + C + SRSD + C:SRSD	6	−16.310	47.330	2.466	.019
A + SRM + SRSD	5	−17.808	47.491	2.627	.017
G + HD + SRSD	6	−16.411	47.532	2.668	.017
A + C + SRM + SRSD	6	−16.462	47.633	2.770	.016
A + C + G + SRSD	7	−14.959	47.651	2.787	.016
A + C + G + HD + SRSD	8	−13.386	47.738	2.875	.015
A + C + G + HD	7	−15.019	47.772	2.908	.015
A	3	−20.587	47.879	3.015	.014
G + SRSD	5	−18.026	47.927	3.064	.014
A + C + HD + SRSD + C:HD	7	−15.147	48.028	3.164	.013
A + C + HD + SRSD + C:SRSD	7	−15.191	48.115	3.251	.013
C + G + HD + C:HD	7	−15.236	48.206	3.342	.012
SRSD	3	−20.779	48.263	3.400	.012
A + G + HD + SRM + SRSD	8	−13.666	48.298	3.435	.011
A + HD + SRM + SRSD	6	−16.838	48.386	3.522	.011
A + C + G + HD + C:G	9	−12.141	48.711	3.847	.009
A + G + HD + SRM	7	−15.533	48.799	3.935	.009
C	3	−21.092	48.889	4.025	.009
A + C + G + HD + SRSD + C:HD	9	−12.250	48.928	4.064	.008
A + C + SRM	5	−18.567	49.009	4.145	.008
C + G + HD + C:G	8	−14.026	49.018	4.154	.008
A + C + G + HD + SRSD + C:SRSD	9	−12.313	49.054	4.190	.008
A + C + G	6	−17.209	49.127	4.263	.008
A + G + SRM + SRSD	7	−15.701	49.136	4.273	.008
A + G	5	−18.644	49.162	4.298	.007
A + C + HD + SRM + SRSD	7	−15.724	49.182	4.318	.007
Null model	2	−22.441	49.224	4.360	.007
C + SRSD	4	−20.041	49.294	4.431	.007
A + C + HD + SRM + C:HD	7	−15.792	49.316	4.453	.007
HD	3	−21.316	49.338	4.474	.007
C + G + HD	6	−17.345	49.401	4.537	.007
G	4	−20.154	49.519	4.656	.006
C + HD + C:HD	5	−18.832	49.539	4.675	.006
HD + SRSD	4	−20.170	49.553	4.689	.006
A + HD + SRM	5	−18.890	49.654	4.791	.006
A + SRM	4	−20.267	49.745	4.881	.006
A + C + HD + SRM	6	−17.531	49.771	4.907	.005
G + HD + SRM	6	−17.576	49.862	4.998	.005
C + G + SRSD	6	−17.579	49.869	5.005	.005
A + C + SRM + SRSD + C:SRSD	7	−16.126	49.986	5.122	.005
C + HD	4	−20.402	50.017	5.153	.005
A + C + HD + SRSD + C:HD + C:SRSD	8	−14.574	50.114	5.250	.005
C + G	5	−19.177	50.228	5.364	.004
A + C + G + SRSD + C:SRSD	8	−14.638	50.242	5.378	.004
C + G + HD + SRSD	7	−16.258	50.250	5.386	.004
C + G + HD + SRSD + C:SRSD	8	−14.706	50.378	5.514	.004
C + G + HD + SRSD + C:HD	8	−14.788	50.542	5.678	.004
G + HD + SRM + SRSD	7	−16.404	50.542	5.678	.004
C + SRSD + C:SRSD	5	−19.351	50.577	5.713	.004
A + C + G + HD + SRM + C:HD	9	−13.100	50.629	5.765	.004
A + C + HD + SRM + SRSD + C:HD	8	−14.836	50.638	5.774	.004
A + C + SRM + SRSD + C:SRM	7	−16.462	50.657	5.793	.004
A + C + G + SRM + SRSD	8	−14.879	50.723	5.859	.003
A + C + HD + SRM + SRSD + C:SRSD	8	−14.883	50.731	5.867	.003
G + SRM + SRSD	6	−18.018	50.745	5.881	.003
SRM + SRSD	4	−20.778	50.768	5.904	.003
A + C + G + HD + SRM + SRSD	9	−13.236	50.901	6.037	.003
A + C + G + HD + SRM	8	−15.013	50.991	6.128	.003
A + G + SRM	6	−18.176	51.062	6.198	.003
SRM	3	−22.187	51.079	6.215	.003
A + C + G + HD + SRSD + C:HD + C:SRSD	10	−11.472	51.091	6.228	.003
C + HD + SRSD	5	−19.621	51.118	6.254	.003
G + SRM	5	−19.709	51.292	6.428	.003
C + SRM	4	−21.079	51.371	6.507	.002
C + G + HD + SRM + C:HD	8	−15.236	51.438	6.574	.002
C + HD + SRSD + C:SRSD	6	−18.372	51.454	6.590	.002
A + C + SRM + C:SRM	6	−18.401	51.512	6.648	.002
C + G + SRSD + C:SRSD	7	−16.950	51.633	6.769	.002
HD + SRM	4	−21.246	51.704	6.840	.002
A + C + G + HD + C:G + C:HD	10	−11.799	51.746	6.882	.002
C + G + HD + SRSD + C:HD + C:SRSD	9	−13.666	51.760	6.896	.002
C + SRM + SRSD	5	−19.966	51.807	6.944	.002
A + C + G + HD + SRSD + C:G	10	−11.839	51.825	6.962	.002
C + G + HD + C:G + C:HD	9	−13.700	51.828	6.964	.002
C + HD + SRSD + C:HD	6	−18.591	51.891	7.027	.002
A + C + G + HD + SRM + C:G	10	−11.891	51.931	7.067	.002
A + C + G + SRM	7	−17.132	51.998	7.134	.002
Full model	14	−10.298	66.857	21.993	.000
